# Parents’ Knowledge and Practices Regarding Children’s Oral Hygiene: A Cross-Sectional Survey

**DOI:** 10.7759/cureus.100792

**Published:** 2026-01-05

**Authors:** Renea Popovic, Stipo Cvitanovic, Inge Sarac, Zvonimir Lukac, Zorana Ivankovic Buljan, Ruzica Zovko

**Affiliations:** 1 Dental Medicine, School of Medicine, University of Mostar, Mostar, BIH; 2 Orthodontics, School of Medicine, University of Mostar, Mostar, BIH; 3 Periodontology, School of Medicine, University of Mostar, Mostar, BIH; 4 Pediatric Dentistry, School of Medicine, University of Mostar, Mostar, BIH

**Keywords:** bosnia and herzegovina, children, cross-sectional study, dental visits, oral hygiene, parental knowledge, tooth brushing

## Abstract

Objective

To assess parents’ knowledge of recommended oral-hygiene practices for children and to describe oral-hygiene behaviors within families (parents and children), as well as factors associated with children’s oral-hygiene outcomes.

Methods

A cross-sectional study was conducted in Mostar (January-June 2025) among 405 parents or guardians of children aged 0-18 years. A structured questionnaire assessed sociodemographic characteristics, oral-hygiene habits, and parental knowledge. The questionnaire was specifically developed for this study and validated through expert review and pilot testing. Descriptive statistics, Spearman’s correlation, Mann-Whitney U, and chi-square tests were used, with statistical significance set at p<0.05.

Results

Most parents (n=367; 90.6%) and children (n=359; 88.6%) brushed their teeth at least twice daily. Only (n=139; 34.3%) of parents began brushing their child’s teeth before the first year of life, and the mean age of the first dental visit was 3.42 years. Although (n=316; 78.2%) of children used age-appropriate toothpaste, adherence to recommended toothpaste use among parents of younger children varied. Children’s oral-hygiene behaviors showed significant associations with maternal education, parents’ frequency of tooth brushing, parental use of oral-hygiene products, and parents’ self-assessed oral-health knowledge (all p<0.05). Parental knowledge and maternal education were the strongest and most consistent predictors.

Conclusion

Despite the high percentage of tooth brushing among children and parents, parents in Bosnia and Herzegovina often fail to follow recommendations for maintaining oral hygiene, such as initiating tooth brushing early, using the proper dosage of toothpaste, and scheduling an early first visit to the dentist. The results highlight the importance of national guidelines and systematic education for parents to enhance children's oral health.

## Introduction

Poor oral health, particularly tooth decay, in early childhood is a significant public health problem [1]. Oral diseases affect the oral health-related quality of life of more than half of children and adolescents worldwide [2]. Low political priority, combined with the failure to integrate oral health into broader health systems, has contributed to its neglect in previous decades [3]. Data from the latest Global Burden of Disease Study indicate that between 1990 and 2019, the number of new cases of untreated primary tooth decay increased by 1.15 billion. Of these, about 62.9 million can be attributed to sociodemographic inequalities [4].

Early childhood caries (ECC) is a disease of deciduous teeth in children under six years of age. ECC is often untreated, causing painful conditions and impairing the overall health, growth, and development of children [5]. The key factors that cause carious lesions in adults and children are similar. Freshly eroded tooth surfaces may have hypoplastic defects that facilitate the accumulation of bacterial biofilms and an increased risk of caries [6]. A literature review conducted in Canada highlighted the main risk factors for caries: enamel hypoplasia, dietary habits, difficulty with brushing, guardian influence, low income, and low level of education. In addition, those who do not have their first dental check-up within 24 months of age appear to be at higher risk of developing carious lesions. Countries in Central and Eastern Europe have shown a high prevalence of tooth decay among children [6].

The risk of developing dental caries in Bosnia and Herzegovina is twice the regional average among 12-year-olds. Similarly, the overall oral health status in Bosnia and Herzegovina is among the worst in Europe [7]. The role of family and community is well established as a key factor in children's oral health. Children usually learn oral health habits from their parents, as parents and guardians are responsible for the care and perception of children's oral health, especially among preschool children [1]. Mechanical removal of dental plaque, if properly implemented, can be the most effective method of maintaining good oral hygiene, reducing caries, and promoting better gingival health [8].

It is recommended that children start brushing their teeth when their first tooth erupts, and their first visit to the dentist should occur no later than one year of age. The Centers for Disease Control and Prevention (CDC) recommends that children start using fluoride toothpaste at age two. Too much fluoride while teeth are developing can result in noticeable changes in the structure of the enamel, such as discoloration and pitting (dental fluorosis). Children under three years of age should use a rice-sized amount of toothpaste, and children over three years of age should use a pea-sized amount (0.25 g) until the age of six, when the swallowing reflex is sufficiently developed to prevent accidental swallowing [9].

The need for this study arose from the fact that, compared to neighboring countries and the European Union, Bosnia and Herzegovina has the worst oral health among children. This cross-sectional study aimed to assess parents’ knowledge of recommended oral-hygiene practices for children and to describe oral-hygiene behaviors within families (parents and children) in Bosnia and Herzegovina, as well as factors associated with children’s oral-hygiene outcomes. The search included databases, Google Scholar, PubMed, Scopus, and Hrčak until June 2025, using the keywords in Croatian and English "Oral hygiene in children", parental knowledge, tooth brushing habits, Bosnia and Herzegovina, independently and in combination with Boolean operators.

The only research that includes a parental perspective is Oral health behavior of nine-year-old children and their parents in Sarajevo (2019), while other papers focus on dental caries, local indicators of oral health, and adolescent attitudes, without a direct analysis of parental knowledge, which plays a key role in maintaining and promoting oral health in children. We hypothesized that parental knowledge, education level, and parents’ own oral-hygiene behaviors would be significantly associated with their children’s oral-hygiene practices.

## Materials and methods

Ethical approval and standard of reporting

This cross-sectional study was approved by the Ethics Committee of the Mostar Health Center (Approval number: 933-37-228/25). Parents were informed about the research process and the conditions of voluntary participation. Written informed consent was obtained from all participants. This cross-sectional survey was conducted from January to June 2025 among parents of preschool and school-age children in Mostar.

The study used a convenience sample of parents who were willing and available to participate at the time of data collection. A total of 700 questionnaires were printed and distributed among parents of preschool and school children, but 405 sufficiently completed questionnaires were collected, which corresponds to a response rate of 57.86%. The remaining questionnaires were not returned or were insufficiently completed for adequate analysis.

The minimum required sample size was calculated using the standard formula for prevalence studies, assuming a 95% confidence level, a 5% margin of error, and a default expected prevalence of 50% - a recommended approach when no prior prevalence data are available (Naing et al.). This calculation yielded a minimum required sample size of 385 participants [10].

Inclusion criteria were that respondents were parents or guardians of children aged 0-18 who voluntarily agreed to participate in the study by completing an anonymous questionnaire. Exclusion criteria were: questionnaires that were largely incomplete and could not be analysed, adults who were not parents or guardians of a child under 18, and ambiguous or contradictory answers that prevented reliable analysis.

Sociodemographic measures

Socioeconomic class was self-assessed by participants and categorized into three groups: lower (financial difficulty or below-average household income), medium (stable average household income), and higher (above-average income or comfortable financial status). Self-assessed oral health was measured using a five-point Likert scale: 1 = very poor, 2 = poor, 3 = moderate, 4 = good, 5 = excellent. Subjective Likert scales for oral-health assessment are routinely used in international epidemiological surveys (WHO Oral Health Surveys, Eurobarometer). These scales intentionally measure perceived rather than clinically defined oral-health status. To limit interindividual variability, all respondents received identical instructions, the variable was treated strictly as ordinal, and non-parametric tests (Spearman’s correlation, Mann-Whitney U, χ²) were applied.

Development and validation of the questionnaire

For the purposes of this study, a questionnaire was developed de novo by the authors, based on relevant literature [6] and international guidelines [European Academy of Paediatric Dentistry (EAPD), CDC] [11-13]. No previously published or proprietary instruments were used. The questionnaire consisted of two sections: the first covered sociodemographic characteristics of parents and children, while the second examined oral-hygiene habits and parental knowledge of recommended practices. The survey was distributed in kindergartens, primary schools, and family medicine or community health centers in Mostar. Parents received the paper-based questionnaire either when bringing or collecting their children from educational institutions or during routine visits to primary healthcare facilities.

The instrument contained 20 items, most of which were closed-ended questions. These items addressed sociodemographic characteristics, oral-hygiene habits of parents and children, and parental knowledge of recommended oral-health guidelines. Since the questionnaire was originally developed in Croatian specifically for this study, no permissions were required for its use. For publication purposes, the questionnaire was translated into English to ensure linguistic clarity. Content validity was confirmed by a three-member expert panel (paediatric dentistry, preventive dentistry, and epidemiology). Pilot testing was conducted among 30 parents who did not participate in the main study, resulting in minor adjustments. The complete questionnaire is provided in Appendices 1-3 (English version) and Appendices 4-6 (Croatian version).

Statistical analysis

Data preparation and coding were performed in Microsoft Excel, while all statistical analyses were conducted using SPSS version 29 (IBM Corp., Armonk, NY). Descriptive statistics (frequencies, percentages, means, and standard deviations) were used to summarise the characteristics of the study sample. Normality of continuous and ordinal variables was assessed using the Shapiro-Wilk test and visual inspection of distribution histograms. Given the ordinal and categorical nature of the variables and their non-parametric distribution of the data, the following statistical methods were applied: Spearman’s rank correlation coefficient to assess associations between ordinal variables; the Mann-Whitney U test to compare differences in ordinal dependent variables between two independent groups; and the chi-square test of independence (χ² test) to examine relationships between categorical variables and differences in response distributions. Response categories were coded numerically in ascending order according to increasing levels of frequency, intensity, or educational attainment, as appropriate for ordinal analyses. All statistical tests were two-tailed, with the significance level set at p<0.05. Results are presented as correlation coefficients (ρ), Mann-Whitney U statistics, and chi-square values (χ²), together with corresponding p-values.

Explanation for the use of Spearman correlation with ordinal categorical variables

Spearman’s rank correlation was used because many questionnaire items were categorical but inherently ordinal in nature. Variables such as brushing frequency, dental visit frequency, and several self-assessment items consisted of ordered response categories (e.g., “never”, “sometimes”, “often”, “always”), which can be meaningfully ranked. Spearman’s correlation does not require normally distributed data and is appropriate for ranked variables. Therefore, all variables with ordered response options were treated as ordinal and analyzed using Spearman’s rank correlation to ensure correct and transparent handling of non-parametric, non-normally distributed data.

## Results

Characteristics of research participants

The questionnaire was completed by 405 parents or guardians of children aged 0-18 years. All surveys were completed in paper format, and the sociodemographic characteristics of the parents or guardians are listed in Table 1. The majority of responses, 248 (61.2%), were completed by mothers. The average age of the child surveyed in the study was 8.36 years with a standard deviation of 4.39 years. Most mothers who participated in the study had a high school diploma, 90 (36.4%), while the largest proportion of fathers reported having a post-secondary vocational education, 55 (34.3%). On a self-assessment scale of oral health knowledge, most parents stated that their knowledge of oral health was moderate, 177 (43.7%). The majority of parents who completed the survey, 286 (71%), resided in urban areas. Furthermore, most parents 321 (79.3%) declared that their socioeconomic status was average (Table 1).

**Table 1:** Sociodemographic characteristics of research participants Socioeconomic class was self-reported and categorized as: lower (financial difficulty or below-average household income), medium (stable average household income), and higher (above-average income or comfortable financial status).

Variable	Category	N=405	N(%)
Sex	Male	157	38.8%
	Female	248	61.2%
	n	405	100%
Child's age	Mean 8.36± SD 4.39 years		
Mother's education level	Primary school	50	20%
	High school	90	36.4%
	Post-secondary vocational education	84	33.9%
	Higher education	18	7.4%
	Doctorate	6	2.2%
	n	248	61.2%
Father's education level	Primary school	19	12.3%
	High school	54	34.1%
	Post-secondary vocational education	55	34.3%
	Higher education	25	16.5%
	Doctorate	4	2.7%
	n	157	38.8%
Oral health self-assessment	Very poor	18	4.4%
	Poor	64	15.8%
	Moderate	177	43.7%
	Good	104	25.7%
	Excellent	42	10.4%
	n	405	100 %
Place of residence	Rural area	117	29.0%
	Urban area	286	71.0%
	n	403	99.51%
Socioeconomic class	Lower	35	8.6%
	Medium	321	79.3%
	Higher	49	12.1%
	n	405	100%

Oral hygiene habits of children and parents

Adherence to oral hygiene habits in children and their parents was investigated based on the 2018 guidelines of the EAPD for children, CDC recommendations, and the 2023 Tooth Brushing Recommendations Through Professional Consensus for the general population.

From this data analysis (Table 2), it follows that 239 (59%) of parents adhere to the recommendation to brush their teeth twice a day, while 128 (31.6%) report brushing their teeth more than twice daily. Parents reported that their children brush their teeth twice daily in 271 (66.9%) of cases. Additionally, 88 (21%) of respondents report brushing their teeth more than twice a day. Parents of children in the 0 to eight-year age group reported in 79 (39.9%) of cases that children should be assisted when brushing their teeth, while in the 0 to two-year group, 29 (63%) of parents stated that their children require assistance with toothbrushing. In the three to eight-year age group, 50 (32.9%) of parents think that children should be helped to brush their teeth.

**Table 2:** Oral hygiene practices of children and parents Subgroup analyses were performed on participants with complete data for the corresponding variables; therefore, sample sizes may vary between tables and within age-stratified subgroups.

Oral hygiene practices in children and parents		Results N=405	n percentage%
How often do parents brush their teeth daily?	Once a day	37	9.1%
	Twice a day	239	59.0%
	More than twice a day	128	31.6%
	Sometimes	1	0.2%
	n	405	100%
How often does your child brush his/her teeth?	Once a day	42	10.4%
	twice a day	271	66.9%
	More than twice a day	88	21.7%
	Sometimes	4	1.0%
	n	405	100%
Do you think a child should be assisted to brush his/her teeth (0-8 years old)?	Yes	79	39.9%
	No	58	29.3%
	Sometimes	61	30.8%
	n	198	48.89%
Do you think a child should be assisted to brush his/her teeth (0-2 years)?	Yes	29	63.0%
	No	7	15.2%
	Sometimes	10	21.7%
	n	46	11.36%
Do you think a child should be assisted to brush his/her teeth (3-8 years old)?	Yes	50	32.9%
	No	51	33.6%
	Sometimes	51	33.6%
	n	152	37.53%
How often do you visit the dentist?	When there is a problem	146	36.0%
	Once a year	133	32.8%
	Twice a year	126	31.1%
	n	405	100%
How often does your child visit the dentist?	When there is a problem	115	28.6%
	Once a year	107	26.6%
	Twice a year	180	44.8%
	n	402	99.26%
How your child rinses his/her mouth after brushing (0-8 years)	Under strict supervision, with little water	28	19.4%
	They wash themselves.	35	24.3%
	I don't care how they rinse their teeth.	18	12.5%
	They don't rinse their teeth afterwards.	23	16.0%
	Sometimes I pay attention to how they rinse their teeth.	30	20.8%
	They rinse their teeth with a larger amount and more often	10	6.9%
	n	144	35.56%
How your child rinses his/her mouth after brushing (0-2 years)	Under strict supervision, with little water	12	27.3%
	They rinse themselves.	3	6.8%
	I don't care how they rinse their teeth.	5	11.4%
	They don't rinse their teeth afterwards.	14	31.8%
	Sometimes I pay attention to how they rinse their teeth.	10	22.7%
	n	44	10.86%
How your child rinses his/her mouth after brushing (2-8 years)	Under strict supervision, with little water	16	17.2%
	They rinse themselves.	26	28.0%
	I don't care how they rinse their teeth.	13	14.0%
	They don't rinse their teeth afterwards.	9	9.7%
	Sometimes I pay attention to how they rinse their teeth.	20	21.5%
	They rinse their teeth with more water and more often	9	9.7%
	n	93	22.96%
At what age did you start brushing your child's teeth?	Less than a year	139	34.3%
	Less than two years	186	45.9%
	Less than three years	54	13.3%
	Less than four years	19	4.7%
	Less than five years	7	1.7%
	n	405	100%
Parental participation in brushing children's teeth (0-8 years)	Parents brush their child's teeth every time	17	12.1%
	I actively participate in brushing teeth.	67	47.5%
	I sometimes participate in brushing teeth.	34	24.1%
	Child brushes teeth independently	17	12.1%
	I never participate in that.	6	4.3%
	n	141	34.81%
Parental participation in brushing children's teeth (0-2 years)	Parents brush their child's teeth every time	15	34.1%
	I actively participate in brushing teeth	24	54.5%
	I sometimes participate in brushing teeth	5	11.4%
	Child brushes teeth independently	0	0%
	I never participate in that.	0	0%
	n	44	10.86%
Parental participation in brushing children's teeth (3-8 years)	Parents brush their child's teeth every time	2	2.0%
	I actively participate in brushing teeth.	42	42.8%
	I sometimes participate in brushing teeth.	28	28.6%
	Child brushes teeth independently	17	17.3%
	I never participate in that.	9	9.1%
	n	98	24.20%
How often do your children consume sweets (total)	Never	17	4.3%
	Once or less per day	191	48.0%
	Two or more times a day	190	47.7%
	n	398	98.27%
For children up to 2 years old	Never	6	13.3%
	Once or less per day	24	53.3%
	Two or more times a day	15	33.3%
	n	45	11.11%
For children 3-8 years	Never	10	6.8%
	Once or less per day	80	54.1%
	Two or more times a day	58	39.2%
	n	148	36.54%
Do you put toothpaste on your child's teeth? (0-8 years)	Yes	53	37.3%
	No	29	20.4%
	Sometimes	60	43.3%
Does your child use age-appropriate toothpaste?	Yes	316	78.2%
	I don't know.	57	14.1%
	It doesn't matter what paste he/she uses.	31	7.7%
	n	404	99.75%
			Products used by parents in oral hygiene	Just a toothbrush	6	1.4%
Just dental floss	4	1.0%	
Just a mouthwash	0	0%	
Just a toothpaste	0	0	
Interdental brushes only	2	0.5%	
Toothbrush and toothpaste	90	22.2%	
Toothbrush, toothpaste and floss	169	41.7%	
Brush, toothpaste, floss and interdental brushes	99	24.4%	
Toothbrush, toothpaste, floss, interdental brushes and mouthwash	35	8.6%	
n	405	100%	
Reason for a child's first visit to the dentist	Toothache	81	20.4%
	Preventive examination	214	53.9%
	Familiarity with the surroundings	102	25.7%
	n	397	98.02%
At what age did you take your child to his/her first dental check-up?	Mean 3.42 SD 1.85	405	100%
What products does the child use for oral hygiene?	Just a toothbrush	2	0.49%
	Just dental floss	0	0%
	Just a mouthwash	0	0%
	Just a toothpaste	0	0%
	Interdental brushes	0	0%
	Toothbrush and toothpaste	226	55.8%
	Toothbrush, toothpaste and dental floss	125	30.84%
	Toothbrush, toothpaste, dental floss and interdental brushes	40	9.8%
	Toothbrush, toothpaste, dental floss, interdental brushes and mouthwash	12	2.9%
	n	405	100%

Most parents 146 (36.0%) visit the dentist only when a problem arises. Parents stated in 180 (44.8%) of cases that they take their children for dental visits. Most parents 35 (24.3%) in the 0 to eight-year age group reported that their children rinse their mouths with water after brushing. In the 0 to two-year group, 14 (31.8%) of parents stated that their children do not rinse after brushing, while in the two to eight-year group, 26 (28%) of parents reported that their children rinse with water after brushing. In the 0 to two-year age group, parents stated that in 24 (54.5%) of cases, they actively participate in brushing their children’s teeth, while in the two to eight-year group, parents actively participate in 42 (42.8%) of cases. Children consume sweets once or less per day in 191 (48%) of cases. For individual groups, for children up to two years old, in 24 (53.3%) of cases, they consume sweets once or less per day. In the age group of three to eight years, 80 (54.1%) of children eat sweets once or less per day. In the age group 0 to eight years, 60 (43.3%) of parents stated that they sometimes apply toothpaste to their child's toothbrush. In the 0 to two-year group, this practice is dominant. In the analysis of the answers to the question of when they started brushing their children's teeth, 186 (45.9%) of cases indicated that they began brushing before the age of two, while 139 (34.3%) reported that they began before the age of one. The majority of parents 169 (41.7%) use a toothbrush, toothpaste, and dental floss to maintain oral hygiene. A toothbrush and toothpaste alone are used by 226 (55.8%) of children. The average age of a child's first visit to the dentist was 3.42 years, with a standard deviation of 1.85. The reason for the first visit in 214 (53.9%) of cases was a preventive examination (Table 2).

Bivariate analysis of selected socioeconomic factors in relation to oral hygiene habits in children and parents

Significant associations were observed between several parental factors and children’s oral-health behaviors (Table 3). The frequency of brushing a child’s teeth was significantly associated with mothers’ level of education (p<0.001), parents’ self-assessed oral-health knowledge (p<0.001), parents’ brushing frequency (p<0.001), the number of oral-hygiene products parents use (p<0.001), and parental dental-visit frequency (p<0.001). No significant difference was found for place of residence (p=0.292). Choosing age-appropriate toothpaste for children was significantly associated with parental oral-health knowledge (p<0.001) and parents’ tooth-brushing frequency (p<0.001). It was also associated with parental use of oral-hygiene products (p=0.002) and the frequency of dental visits (p=0.018). 

**Table 3:** Bivariate analysis of selected socioeconomic factors in relation to oral hygiene habits in children and parents p<0.05 was considered statistically significant

Outcome (Dependent variable)	Independent variable	Test used	Statistic	p-value
Frequency of brushing a child’s teeth	Mother’s education	Spearman ρ	0.185	<0.001
	Father’s education	Spearman ρ	0.092	0.065
	Economic status	Spearman ρ	0.068	0.171
	Parental oral-health knowledge	Spearman ρ	–0.240	<0.001
	Place of residence	Mann–Whitney U	15802.5	0.292
	Parent brushing frequency	Spearman ρ	0.451	<0.001
	Parent oral-hygiene products	Spearman ρ	0.177	<0.001
	Parent dental-visit frequency	Spearman ρ	0.235	<0.001
Is the toothpaste age-appropriate?	Mother’s education	χ²	11.866	0.457
	Father’s education	χ²	5.40	0.988
	Economic status	χ²	9.85	0.131
	Parental oral-health knowledge	χ²	56.05	<0.001
	Place of residence	χ²	5.97	0.427
	Parent brushing frequency	χ²	34.63	<0.001
	Parent oral-hygiene products	χ²	44.95	0.002
	Parent dental visits	χ²	15.30	0.018
Parental participation in brushing child's teeth (0–8 yrs)	Mother’s education	Spearman ρ	–0.148	0.003
	Father’s education	Spearman ρ	0.020	0.686
	Economic status	Spearman ρ	–0.014	0.779
	Parental oral-health knowledge	Spearman ρ	0.129	0.009
	Place of residence	Mann–Whitney U	17145.0	0.677
	Parent brushing frequency	Spearman ρ	0.018	0.722
	Parent oral-hygiene products	Spearman ρ	–0.122	0.014
	Parent dental visits	Spearman ρ	–0.067	0.181
Parental participation (0–2 yrs)	Mother’s education	χ²	20.927	<0.001
	Father’s education	χ²	6.92	0.226
	Economic status	χ²	5.95	0.051
	Parental oral-health knowledge	χ²	37.45	<0.001
	Place of residence	χ²	1.16	0.560
	Parent brushing frequency	χ²	3.81	0.283
	Parent oral-hygiene products	χ²	10.36	0.169
	Parent dental visits	χ²	20.53	<0.001
Child sweet consumption (total)	Mother’s education	Spearman ρ	–0.160	0.001
	Father’s education	Spearman ρ	0.001	0.979
	Economic status	Spearman ρ	–0.201	<0.001
	Parental oral-health knowledge	Spearman ρ	0.109	0.029
	Place of residence	Mann–Whitney U	17504.5	0.413
	Parent brushing frequency	Spearman ρ	–0.067	0.175
	Parent oral-hygiene products	Spearman ρ	–0.111	0.025
	Parent dental visits	Spearman ρ	–0.051	0.307
Number of oral-hygiene products child uses	Mother’s education	Spearman ρ	–0.036	0.473
	Father’s education	Spearman ρ	0.058	0.245
	Economic status	Spearman ρ	0.109	0.028
	Parental oral-health knowledge	Spearman ρ	–0.119	0.016
	Place of residence	Mann–Whitney U	18606.0	0.051
	Parent brushing frequency	Spearman ρ	0.128	0.010
	Parent oral-hygiene products	Spearman ρ	0.357	<0.001
	Parent dental visits	Spearman ρ	0.147	0.003
Age of first dental visit (child)	Mother’s education	Spearman ρ	–0.371	<0.001
	Father’s education	Spearman ρ	–0.117	0.019
	Economic status	Spearman ρ	–0.147	0.003
	Parental oral-health knowledge	Spearman ρ	0.200	<0.001
	Place of residence	Mann–Whitney U	17416.0	0.513
	Parent brushing frequency	Spearman ρ	–0.023	0.644
	Parent oral-hygiene products	Spearman ρ	–0.180	<0.001
	Parent dental visits	Spearman ρ	–0.139	0.005

Parental participation in brushing their child’s teeth was significantly associated with maternal education level (p=0.003), parental oral-health knowledge (p=0.009), and the number of oral-hygiene products used by the child (p=0.014). No significant associations were observed with socioeconomic status or place of residence.

For children aged 0 to two years, significant differences were found according to maternal education (p<0.001), parents’ oral-health knowledge (p<0.001), and parental dental-visit frequency (p<0.001).

Children of mothers with higher education (p=0.001) and from families with higher socioeconomic status (p<0.001), as well as those whose parents used a greater number of oral-hygiene products (p=0.025), consumed sweets less frequently between meals. The number of oral-hygiene products the child uses was significantly associated with socioeconomic status (p=0.028), parental oral-health knowledge (p=0.016), parental brushing frequency (p=0.010), the number of products parents use (p<0.001), and parental dental-visit frequency (p=0.003).

The timing of the child’s first dental check-up was significantly associated with maternal education, parents’ self-assessed oral-health knowledge, socioeconomic status, both the use of oral-hygiene products and the frequency of dental visits by parents (all p<0.05). Graphical representations of all bivariate associations presented in Table 3 are provided in Appendices 7-13 to facilitate visual interpretation of the results.

## Discussion

Epidemiological data on the incidence of cavities in children in Bosnia and Herzegovina are very scarce. Given the lack of an adequate national database or a national program for the treatment of cavities in children, we cite data from the World Health Organization (WHO), which reports that the prevalence of untreated caries in children aged one to nine years in Bosnia and Herzegovina is 52.7% [14].

According to various studies, the prevalence of childhood caries in Bosnia and Herzegovina ranges between 87.61% and 89.1% [15,16]. Given that there are almost no significant regional or national actions aimed at promoting oral health in Bosnia and Herzegovina, our research focused on the knowledge and habits of parents in maintaining oral hygiene. It has been known for a long time that parents and guardians play a key role in maintaining oral hygiene in children and promoting proper nutrition [17-19].

Compliance with recommendations

Given that there are no national guidelines for maintaining oral hygiene in children and adults, we investigated adherence to certain recommendations by the American Dental Association and to the 2024 consensus-based guidelines (Glenny et al. Development of Tooth Brushing Recommendations Through Professional Consensus) [20].

In practice, our findings show that the vast majority of parents and children do not adhere to the recommendations for maintaining oral hygiene. Parents overwhelmingly adhere to recommendations on the frequency of tooth brushing, with 367 (90.6%) of parents brushing their teeth two or more times a day, while 359 (88.6%) of children brush their teeth two or more times a day. What is concerning is that in the category of children up to eight years of age, 61 (30.8%) of parents think that their child should not be assisted in brushing his/her teeth, while 58 (29.3%) of parents think that they should not be assisted at all. Even more concerning is that 7 (15.25%) of parents of children up to two years of age do not think that their child should be assisted in brushing his/her teeth, and 10 (21.7%) of them think that they should sometimes assist their child in brushing his/her teeth. Parents or guardians should continue to help or supervise tooth brushing until the child can brush his/her teeth effectively on their own [20]. According to various studies and guidelines, the period during which partial or complete supervision of brushing a child's teeth is required is six to eight years.

It should also be noted that most parents visit the dentist when there is a problem and do not go for recommended preventive check-ups. The vast majority of parents stated that their child visits the dentist once or twice a year. Also, only 23 (16%) of parents of children under the age of eight follow the recommendation not to rinse their mouth after brushing their teeth, while 14 (31.8%) of children under the age of two do not rinse their mouth after brushing.

This study found that only 34% of parents took their child to the dentist for the first time before the age of one. The vast majority of parents, 316 (78.2%), reported that they make sure that their child has age-appropriate toothpaste. Another troubling fact is that only 139 (34.3%) of parents started brushing their children's teeth when they were less than a year old. According to WHO recommendations, it is advisable to start brushing a child's teeth when the first tooth erupts, at an average age of six months [14]. This research has unequivocally shown that parents' habits, such as their own tooth brushing, significantly influence children's habits and behavior.

The results presented in this paper show substantial differences in parents' knowledge of oral health and oral hygiene habits, which, in our opinion, is a consequence of the lack of any significant health policy at the national level to educate parents and children. It would be important to focus attention on parents and to encourage parental education because we know that parents play a key role in maintaining oral hygiene in children. Astrom and Jakobsen reported that parents' behavior towards oral health is a model that their children copy [21]. In this bivariate analysis, several parental factors demonstrated significant associations with children’s oral-hygiene behaviours, most notably maternal education and parents’ self-assessed socioeconomic status. Additionally, the frequency of tooth brushing by parents and the amount of products that parents use in oral hygiene were highlighted. It has also been shown that the more often parents visit the dentist, the more often their children visit the dentist.

This study, as well as the study by Kuter et al., shows that there is a relationship between the level of education of mothers and the habits of brushing their children's teeth [22]. Goldenfum et al. also reported that the frequency of brushing children's teeth increased to three times a day with a higher level of maternal education. Another study reported that a higher level of maternal knowledge about oral health was associated with brushing their children's teeth twice a day [23].

Kuter et al. also found a positive correlation between mothers' level of education and the frequency of brushing their children's teeth. As mothers' level of education increased, so did their children's frequency of brushing their teeth. [22] This study found that the most important factors for adequate oral hygiene in children were the mother's knowledge of oral health, socioeconomic status, and the mother's level of education. In the study, place of residence did not play a significant role in the oral habits of children and parents. It was also shown that the father's education, except for the frequency of candy consumption, did not play a significant role in children's oral hygiene.

Also, self-assessment of oral health did not correlate with oral hygiene habits in children. This study, as well as similar studies in Bosnia and Herzegovina [7] and the European Union [24-26], showed that parents do not have enough knowledge about oral hygiene. In Bosnia and Herzegovina, in particular, compared to other studies, it is emphasized that children visit the dentist when there is a problem, and not for a preventive examination, that children come to the dentist for the first examination later, and that oral hygiene maintenance in children is started later. Significant socioeconomic differences in Bosnia and Herzegovina affect the availability of products for maintaining oral hygiene.

We also emphasize again that almost all European Union countries have a national policy on children's oral health, while in Bosnia and Herzegovina, there is no unified national program for oral hygiene for either children or adults [27]. In our opinion, special attention should be paid to educating parents about oral health. We believe that it is urgent to create guidelines for maintaining oral hygiene in children and adults in Bosnia and Herzegovina and to intensify health policy in this direction based on already known, proven public policies in the European Union.

Limitations

This study has several limitations. First, its cross-sectional design prevents the establishment of causality between parental factors and children’s oral-hygiene outcomes. Second, all variables were self-reported, which introduces recall and social-desirability bias. A small proportion of individual items contained missing data (e.g., place of residence and dietary habits). However, the overall level of missing responses was low and handled using pairwise deletion, which is appropriate for cross-sectional survey data. Third, the sample was collected from a single geographic region, which may limit generalizability. Finally, although the questionnaire underwent expert review and pilot testing, full psychometric validation (e.g., test-retest reliability or internal consistency metrics) was not performed.

## Conclusions

This study demonstrated that parents in Bosnia and Herzegovina lack sufficient knowledge of recommended oral-hygiene practices and that common mistakes in everyday oral care are frequent. A substantial proportion of children begin tooth brushing later than recommended, toothpaste is often inadequately dosed, and the first dental examination typically occurs after the age of three, frequently prompted by a toothache rather than by preventive care.

Maternal education and parents’ self-assessed oral-health knowledge emerged as the most consistent predictors of children’s oral-hygiene behaviors, while socioeconomic status and parents’ own hygiene habits were associated with several, but not all, oral-health outcomes. Given the high prevalence of dental caries among children in Bosnia and Herzegovina, these findings underscore the urgent need for national oral-health guidelines, earlier first dental visits, and systematic parental education programs aimed at improving children’s oral-health outcomes.

## Figures and Tables

**Figure 1:**
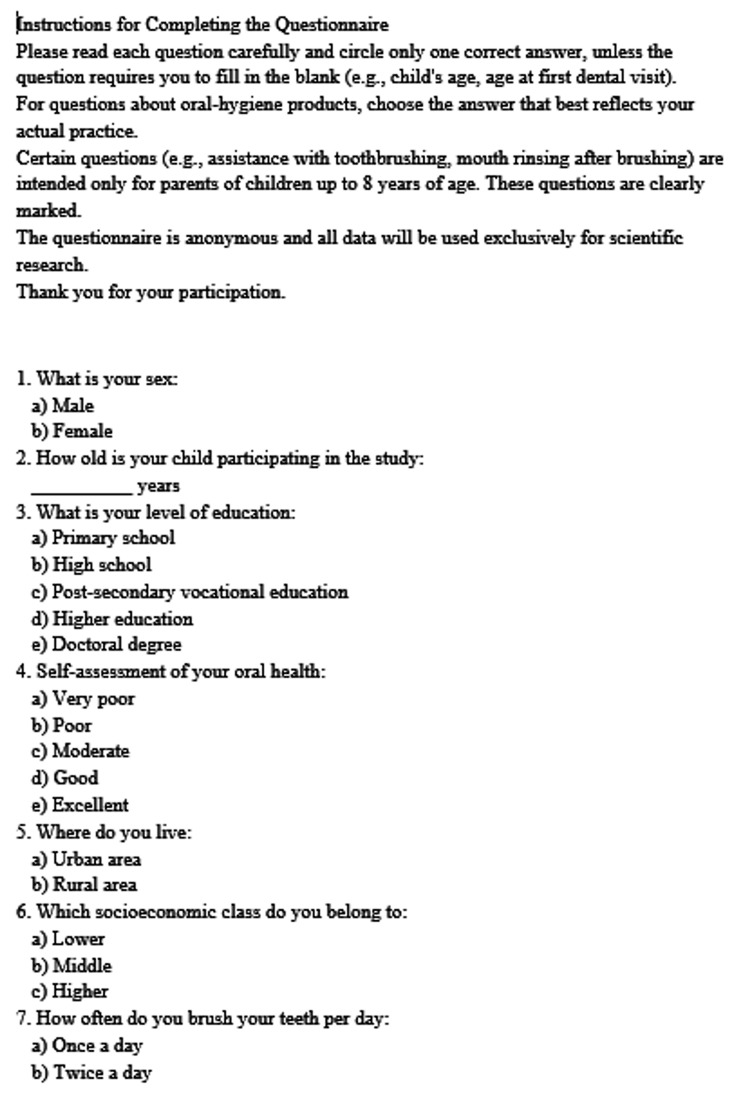
Questionnaire (English version) Part One

**Figure 2:**
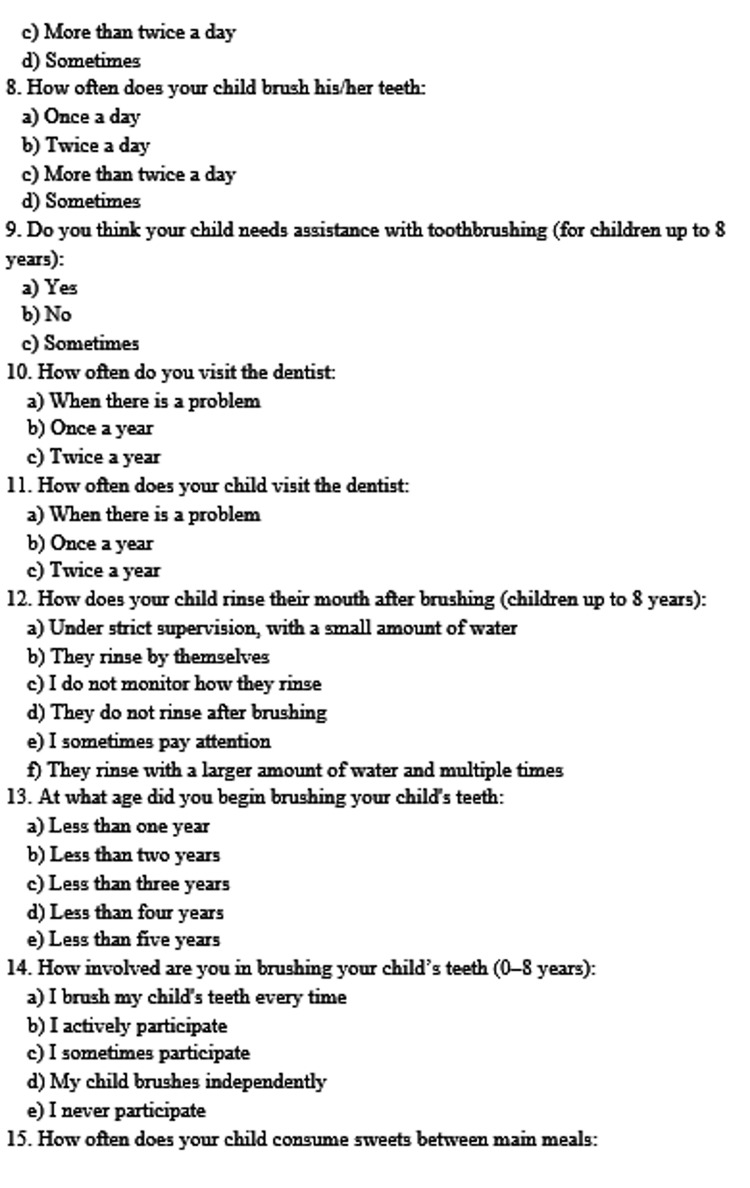
Questionnaire (English version) Part Two

**Figure 3:**
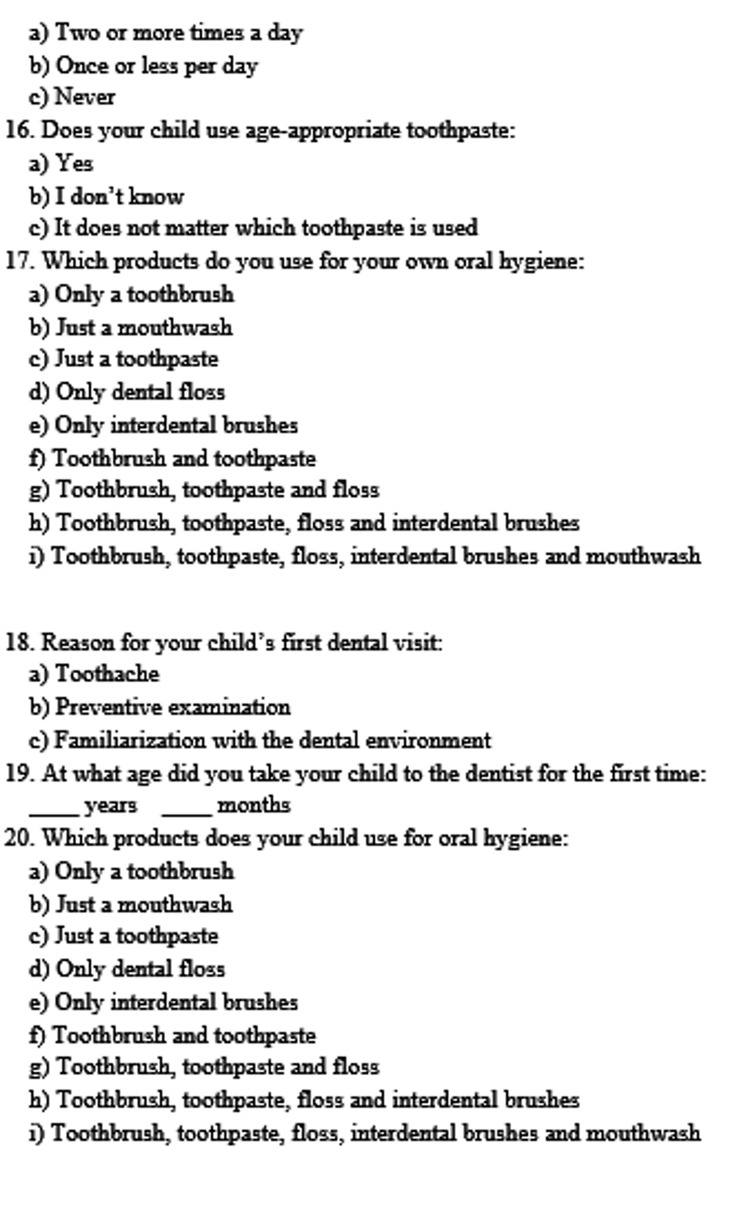
Questionnaire (English version) Part Three

**Figure 4:**
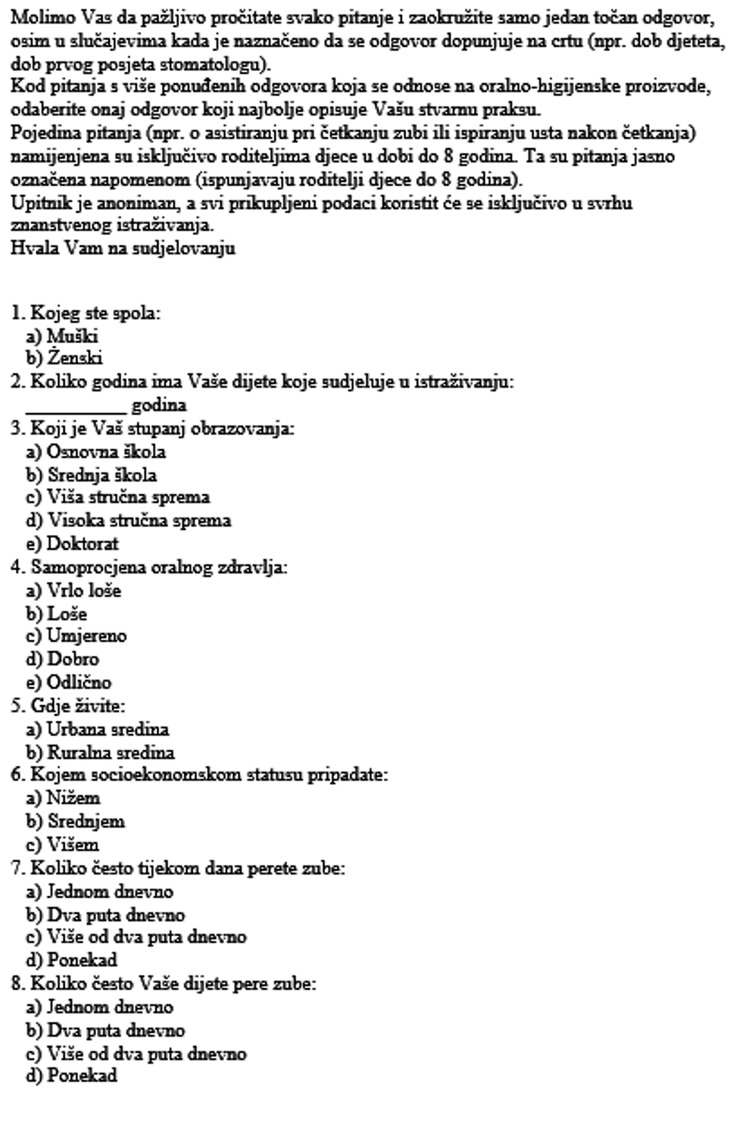
Questionnaire (Croatian version) Part One

**Figure 5:**
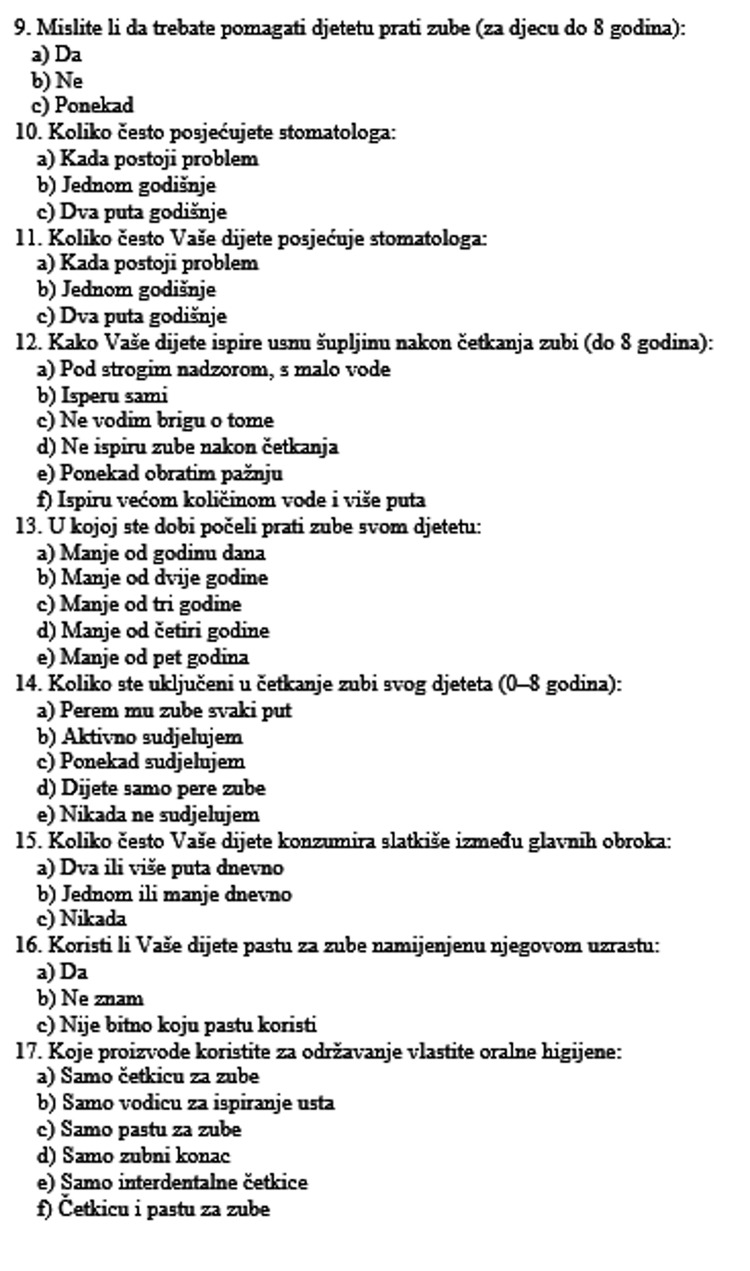
Questionnaire (Croatian version) Part Two

**Figure 6:**
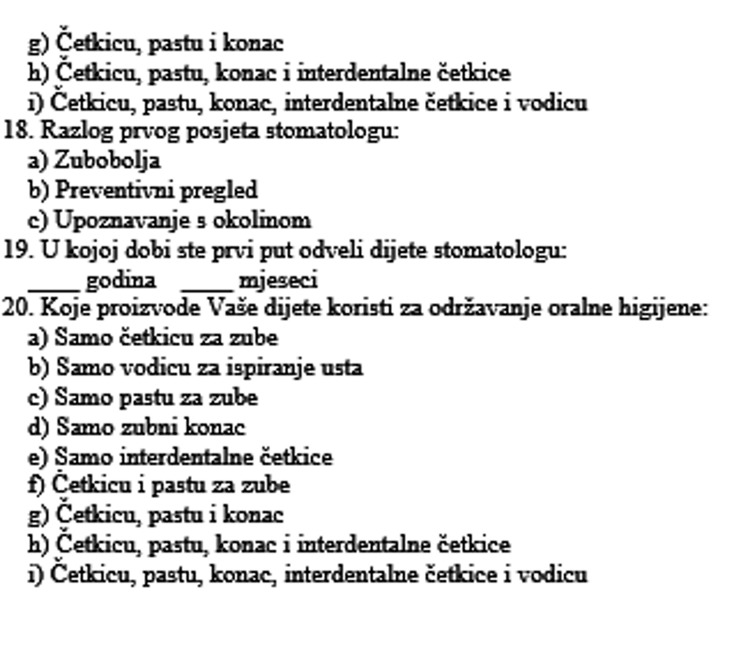
Questionnaire (Croatian version) Part Three

**Figure 7:**
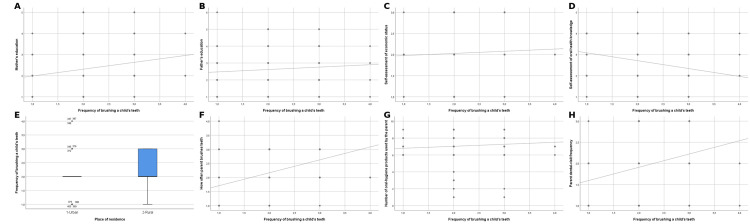
Presents the associations between parental characteristics and the frequency of brushing a child’s teeth. Figure 7A shows the association between a mother’s education level and the frequency of brushing a child’s teeth. A weak but statistically significant positive correlation was observed (Spearman’s ρ=0.185, p<0.001). Figure 7B illustrates the relationship between father’s education and the frequency of brushing a child’s teeth; no statistically significant correlation was found (Spearman’s ρ=0.092, p=0.065). Figure 7C depicts the association between self-assessed economic status and the frequency of brushing a child’s teeth, showing no statistically significant correlation (Spearman’s ρ=0.068, p=0.171). Figure 7D shows the relationship between parental oral-health knowledge and the frequency of brushing a child’s teeth, demonstrating a statistically significant weak negative correlation (Spearman’s ρ=−0.240, p<0.001). Figure 7E compares the frequency of brushing a child’s teeth between urban and rural residents. No statistically significant difference was observed between the two groups (Mann–Whitney U=15,802.5, p=0.292). Figure 7F illustrates the association between parental toothbrushing frequency and the frequency of brushing a child’s teeth, showing a moderate positive correlation (Spearman’s ρ=0.451, p<0.001). Figure 7G presents the relationship between the number of oral-hygiene products used by the parent and the frequency of brushing a child’s teeth, demonstrating a weak but statistically significant positive correlation (Spearman’s ρ=0.177, p<0.001). Figure 7H shows the association between parental dental-visit frequency and the frequency of brushing a child’s teeth, with a weak but statistically significant positive correlation (Spearman’s ρ=0.235, p<0.001).

**Figure 8:**
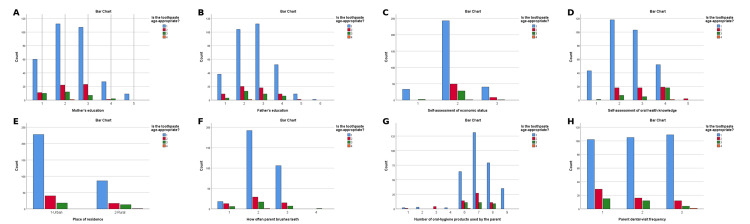
Associations between parental characteristics and the use of age-appropriate toothpaste for children Figure 8 presents the associations between parental characteristics and whether an age-appropriate toothpaste is used for the child. Figure 8A shows the association between mother’s education level and the use of age-appropriate toothpaste; no statistically significant association was observed (χ²=11.87, p=0.457). Figure 8B illustrates the relationship between father’s education and the use of age-appropriate toothpaste, with no statistically significant association detected (χ²=5.40, p=0.988). Figure 8C depicts the association between self-assessed economic status and the use of age-appropriate toothpaste, showing no statistically significant association (χ²=9.85, p=0.131). Figure 8D shows the relationship between parental oral-health knowledge and the use of age-appropriate toothpaste, demonstrating a statistically significant association (χ²=56.05, p<0.001). Figure 8E compares the use of age-appropriate toothpaste between urban and rural residents; no statistically significant difference was observed between the two groups (χ²=5.97, p=0.427). Figure 8F illustrates the association between parental toothbrushing frequency and the use of age-appropriate toothpaste, revealing a statistically significant association (χ²=34.63, p<0.001). Figure 8G presents the relationship between the number of oral-hygiene products used by the parent and the use of age-appropriate toothpaste, showing a statistically significant association (χ²=44.95, p=0.002). Figure 8H shows the association between parental dental-visit frequency and the use of age-appropriate toothpaste, with a statistically significant association observed (χ²=15.30, p=0.018).

**Figure 9:**
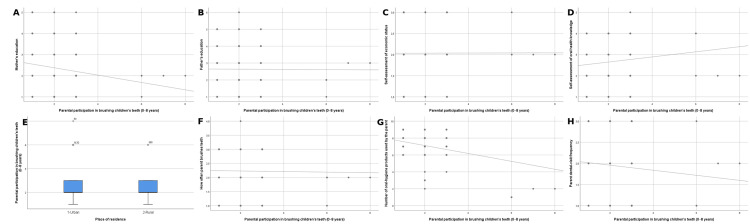
. Associations between parental characteristics and parental participation in brushing children’s teeth (0–8 years) Figure 9 presents the associations between parental characteristics and parental participation in brushing children’s teeth among children aged 0–8 years. Figure 9A shows the association between mother’s education level and parental participation in brushing children’s teeth, demonstrating a weak but statistically significant negative correlation (Spearman’s ρ=−0.148, p=0.003). Figure 9B illustrates the relationship between father’s education and parental participation in brushing children’s teeth; no statistically significant correlation was observed (Spearman’s ρ=0.020, p=0.686). Figure 9C depicts the association between self-assessed economic status and parental participation in brushing children’s teeth, with no statistically significant correlation detected (Spearman’s ρ=−0.014, p=0.779). Figure 9D shows the relationship between parental oral-health knowledge and parental participation in brushing children’s teeth, revealing a weak but statistically significant positive correlation (Spearman’s ρ=0.129, p=0.009). Figure 9E compares parental participation in brushing children’s teeth between urban and rural residents; no statistically significant difference was observed between the two groups (Mann–Whitney U=17,145.0, p=0.677). Figure 9F illustrates the association between parental toothbrushing frequency and parental participation in brushing children’s teeth, showing no statistically significant correlation (Spearman’s ρ=0.018, p=0.722). Figure 9G presents the relationship between the number of oral-hygiene products used by the parent and parental participation in brushing children’s teeth, demonstrating a weak but statistically significant negative correlation (Spearman’s ρ=−0.122, p=0.014). Figure 9H shows the association between parental dental-visit frequency and parental participation in brushing children’s teeth, with no statistically significant correlation observed (Spearman’s ρ=−0.067, p=0.181).

**Figure 10:**
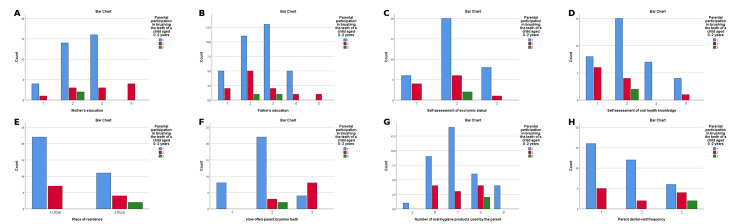
Associations between parental characteristics and parental participation in brushing children’s teeth (0–2 years) Figure 10 presents the associations between parental characteristics and parental participation in brushing the teeth of children aged 0–2 years. Figure 10A shows the association between mother’s education level and parental participation in brushing the teeth of children aged 0–2 years, demonstrating a statistically significant association (χ²=20.927, p<0.001). Figure 10B illustrates the relationship between father’s education and parental participation in brushing the teeth of children aged 0–2 years; no statistically significant association was observed (χ²=6.92, p=0.226). Figure 10C depicts the association between self-assessed economic status and parental participation in brushing the teeth of children aged 0–2 years, showing no statistically significant association (χ²=5.95, p=0.051). Figure 10D shows the relationship between parental oral-health knowledge and parental participation in brushing the teeth of children aged 0–2 years, demonstrating a statistically significant association (χ²=37.45, p<0.001). Figure 10E compares parental participation in brushing the teeth of children aged 0–2 years between urban and rural residents; no statistically significant difference was observed between the two groups (χ²=1.16, p=0.560). Figure 10F illustrates the association between parental toothbrushing frequency and parental participation in brushing the teeth of children aged 0–2 years, with no statistically significant association detected (χ²=3.81, p=0.283). Figure 10G presents the relationship between the number of oral-hygiene products used by the parent and parental participation in brushing the teeth of children aged 0–2 years, showing no statistically significant association (χ²=10.36, p=0.169). Figure 10H shows the association between parental dental-visit frequency and parental participation in brushing the teeth of children aged 0–2 years, demonstrating a statistically significant association (χ²=20.53, p<0.001).

**Figure 11:**
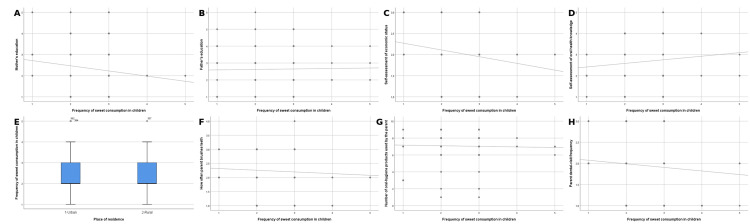
Associations between parental characteristics and the frequency of sweet consumption in children Figure 11 presents the associations between parental characteristics and the frequency of sweet consumption in children. Figure 11A shows the association between mother’s education level and the frequency of sweet consumption in children, demonstrating a weak but statistically significant negative correlation (Spearman’s ρ=−0.160, p=0.001). Figure 11B illustrates the relationship between father’s education and the frequency of sweet consumption in children; no statistically significant correlation was observed (Spearman’s ρ=0.001, p=0.979). Figure 11C depicts the association between self-assessed economic status and the frequency of sweet consumption in children, showing a weak but statistically significant negative correlation (Spearman’s ρ=−0.201, p<0.001). Figure 11D shows the relationship between parental oral-health knowledge and the frequency of sweet consumption in children, demonstrating a weak but statistically significant positive correlation (Spearman’s ρ=0.109, p=0.029). Figure 11E compares the frequency of sweet consumption in children between urban and rural residents; no statistically significant difference was observed between the two groups (Mann–Whitney U=17,504.5, p=0.413). Figure 11F illustrates the association between parental toothbrushing frequency and the frequency of sweet consumption in children, with no statistically significant correlation detected (Spearman’s ρ=−0.067, p=0.175). Figure 11G presents the relationship between the number of oral-hygiene products used by the parent and the frequency of sweet consumption in children, demonstrating a weak but statistically significant negative correlation (Spearman’s ρ=−0.111, p=0.025). Figure 11H shows the association between parental dental-visit frequency and the frequency of sweet consumption in children, with no statistically significant correlation observed (Spearman’s ρ=−0.051, p=0.307).

**Figure 12:**
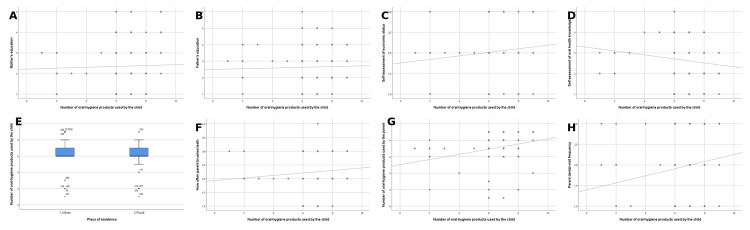
Associations between parental characteristics and the number of oral-hygiene products used by the child Figure 12 presents the associations between parental characteristics and the number of oral-hygiene products used by the child. Figure 12A shows the association between mother’s education level and the number of oral-hygiene products used by the child; no statistically significant correlation was observed (Spearman’s ρ=−0.036, p=0.473). Figure 12B illustrates the relationship between father’s education and the number of oral-hygiene products used by the child, with no statistically significant correlation detected (Spearman’s ρ=0.058, p=0.245). Figure 12C depicts the association between self-assessed economic status and the number of oral-hygiene products used by the child, demonstrating a weak but statistically significant positive correlation (Spearman’s ρ=0.109, p=0.028). Figure 12D shows the relationship between parental oral-health knowledge and the number of oral-hygiene products used by the child, revealing a weak but statistically significant negative correlation (Spearman’s ρ=−0.119, p=0.016). Figure 12E compares the number of oral-hygiene products used by the child between urban and rural residents; no statistically significant difference was observed between the two groups (Mann–Whitney U=18,606.0, p=0.051). Figure 12F illustrates the association between parental toothbrushing frequency and the number of oral-hygiene products used by the child, demonstrating a weak but statistically significant positive correlation (Spearman’s ρ=0.128, p=0.010). Figure 12G presents the relationship between the number of oral-hygiene products used by the parent and the number of oral-hygiene products used by the child, showing a moderate positive correlation (Spearman’s ρ=0.357, p<0.001). Figure 12H shows the association between parental dental-visit frequency and the number of oral-hygiene products used by the child, demonstrating a weak but statistically significant positive correlation (Spearman’s ρ=0.147, p=0.003).

**Figure 13:**
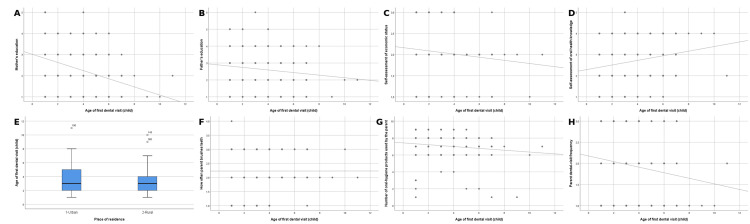
Associations between parental characteristics and the age of the child’s first dental visit Figure 13 presents the associations between parental characteristics and the age of the child’s first dental visit. Figure 13A shows the association between mother’s education level and the age of the child’s first dental visit, demonstrating a moderate and statistically significant negative correlation (Spearman’s ρ=−0.371, p<0.001). Figure 13B illustrates the relationship between father’s education and the age of the child’s first dental visit, showing a weak but statistically significant negative correlation (Spearman’s ρ=−0.117, p=0.019). Figure 13C depicts the association between self-assessed economic status and the age of the child’s first dental visit, demonstrating a weak but statistically significant negative correlation (Spearman’s ρ=−0.147, p=0.003). Figure 13D shows the relationship between parental oral-health knowledge and the age of the child’s first dental visit, revealing a weak but statistically significant positive correlation (Spearman’s ρ=0.200, p<0.001). Figure 13E compares the age of the child’s first dental visit between urban and rural residents; no statistically significant difference was observed between the two groups (Mann–Whitney U=17,416.0, p=0.513). Figure 13F illustrates the association between parental toothbrushing frequency and the age of the child’s first dental visit, with no statistically significant correlation detected (Spearman’s ρ=−0.023, p=0.644). Figure 13G presents the relationship between the number of oral-hygiene products used by the parent and the age of the child’s first dental visit, demonstrating a weak but statistically significant negative correlation (Spearman’s ρ=−0.180, p<0.001). Figure 13H shows the association between parental dental-visit frequency and the age of the child’s first dental visit, revealing a weak but statistically significant negative correlation (Spearman’s ρ=−0.139, p=0.005).

## Appendices

Appendix 1

Appendix 2 

Appendix 3 

Appendix 4 

Appendix 5 

Appendix 6 

Appendix 7 

Appendix 8 

Appendix 9 

Appendix 10 

Appendix 11 

Appendix 12 

Appendix 13 
